# Supplementary material for: Coregulators Reside within Drosophila Ecdysone-Inducible Loci before and after Ecdysone Treatment
Source: Int J Mol Sci. 2023 Jul 24;24(14):11844. doi: 10.3390/ijms241411844 (PMC10380596; doi:10.3390/ijms241411844)

Supplementary figures

Fig. S1

MD plot showing the log-fold change and average abundance of transcripts in S2 cells treated with 20E for 1-hour vs DMSO-treated cells

Significantly up and down DE transcripts are highlighted in red and blue, respectively. Specific transcripts of genes whose transcription increased more than 1.4-fold after 1-hour ecdysone treatment constituted a set of 146 ecdysone-dependent transcripts in S2 cells.

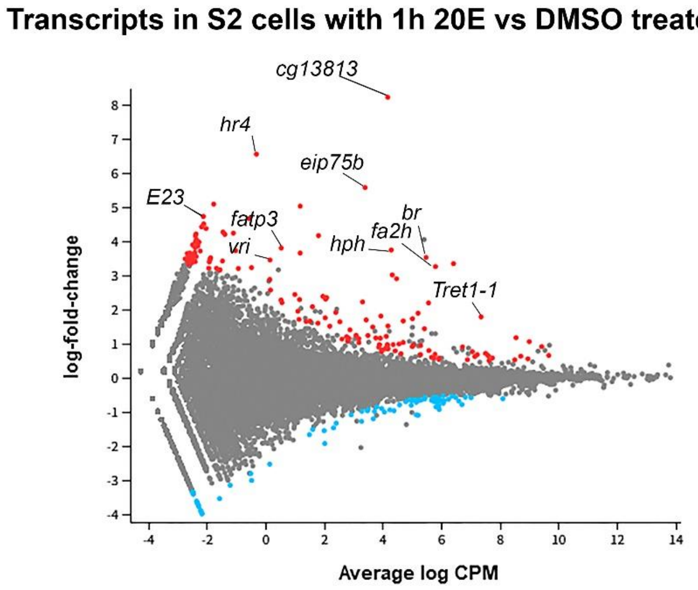

Fig. S2

Binding profiles of RNA polymerase II, FLAG-EcR, various coregulators and the level of H3K27Ac modification at the STARR-Seq enhancers within ecdysone-responsive loci in Drosophila S2 cells

Average distribution of RNA-polymerase II (Rpb3 subunit), FLAG-EcR, CBP/p300/Nejire, Brm, KisL, CHD1, DART1/PRMT1, Gcn5, dSet1, lid, Spt5, PAF1, cdk8 and Histone H3K27Ac modification at the STARR-Seq peaks (N=58) of the genomic loci, whose transcription is induced by 1-hour treatment of Drosophila S2 cells with 20-hydroxyecdysone. ChIP-Seqs and ChIP-MNase Seq for H3K27Ac were performed on Drosophila S2 cells treated with 20-hydroxyecdysone (0.3 µM) for 1 hour "1h 20E" (red graph) or on the sham-treated cells "DMSO" (blue graph). Protein binding levels were calculated as an enrichment (ratio of the corresponding ChIP-Seq signal to the input DNA). Average profiles were calculated as the mean of the protein binding level. The standard error appears on the graphs as a lighter area around the main line of the profiles. The background binding level for the ChIP-Seqs were estimated using the set of 500 randomly chosen regions of the Drosophila genome and provided as a grey transparent area at the averaged plots. ChIP-Seq data on FLAG-EcR (GSE139316), Rpb3 (GSE102520) and CBP/p300/Nejire (GSE156847) binding were loaded from GEO and described previously. ChIP-MNase-Seq data on H3K27Ac are described here for the first time.

STARR-Seq peaks within 20E-inducible loci

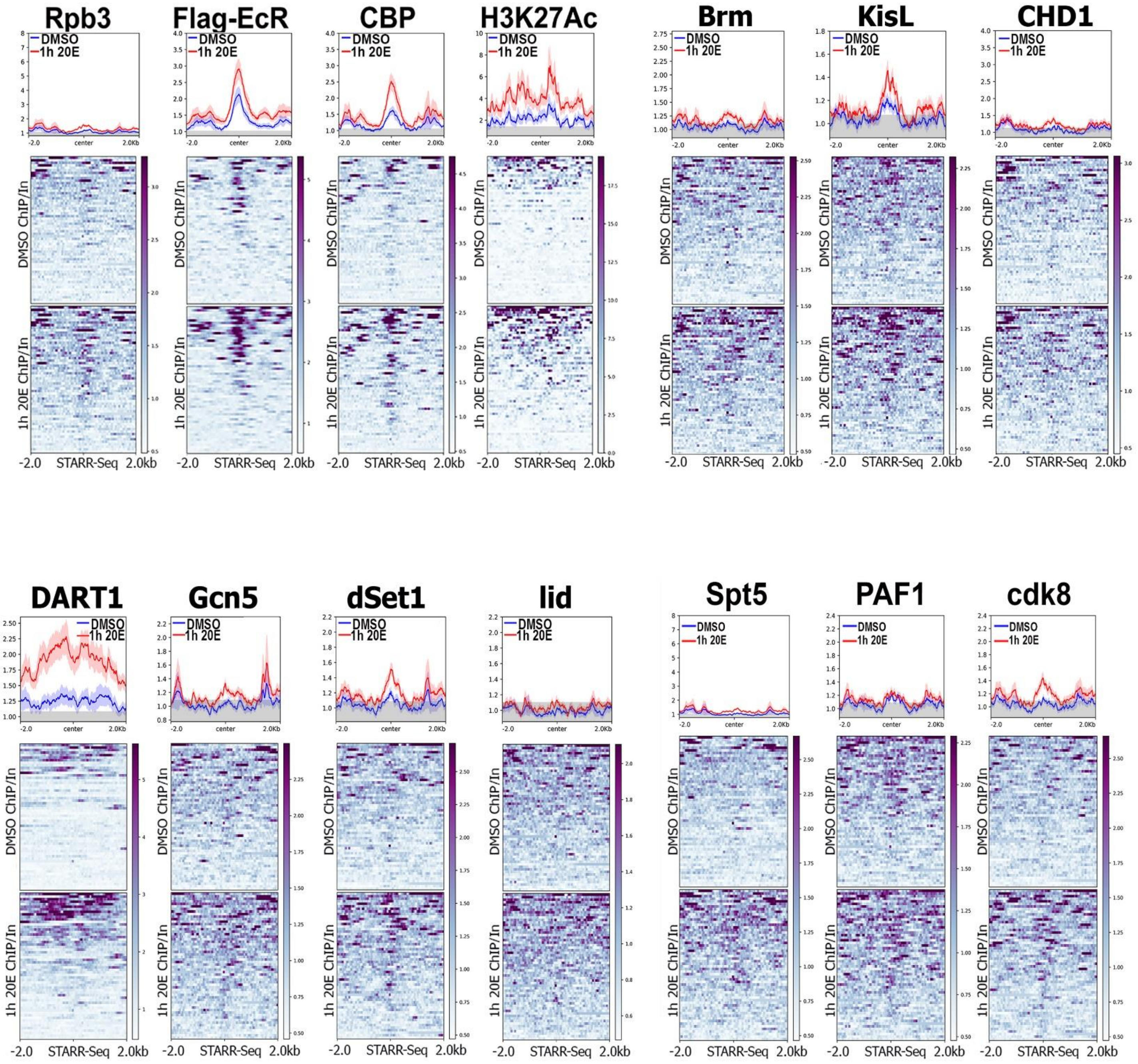

Fig. S3

Many coregulators bind CP190 sites that fall into the primary 20E-responsive loci in S2 cells

Average distribution of Brm, KisL, CHD1, DART1, Gcn5, dSet1, lid/Kdm5, Spt5, PAF1 and cdk8 at the CP190 sites (N=140) of the genomic loci, whose transcription is induced by 1-hour treatment of Drosophila S2 cells with 20-hydroxyecdysone. ChIP-Seqs were performed on Drosophila S2 cells treated with 20-hydroxyecdysone (0.3 µM) for 1 hour “1h 20E” (red graph) or on the sham-treated cells “DMSO” (blue graph). Protein binding levels were calculated as an enrichment (ratio of the corresponding ChIP-Seq signal to the input DNA). Average profiles were calculated as the mean of the protein binding level. The standard error appears on the graphs as a lighter area around the main line of the profiles. The background binding level for the ChIP-Seqs were estimated using the set of 500 randomly chosen regions of the Drosophila genome and provided as a grey transparent area at the averaged plots.

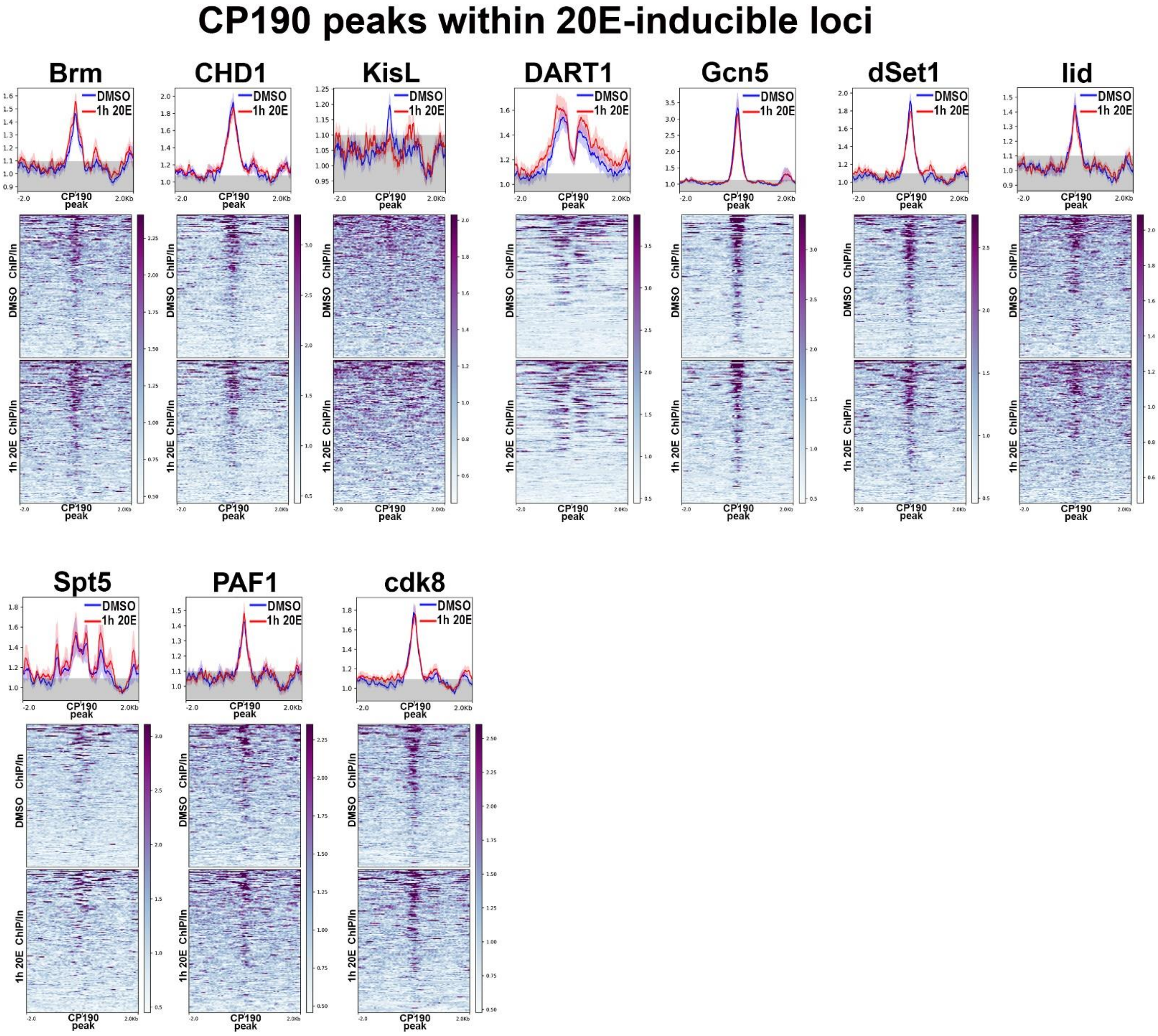

Supplement: Supplementary file 1 [file ijms-24-11844-s001.zip › Supplementary figures.pdf]
